# Influence of respiration on cerebrospinal fluid movement using magnetic resonance spin labeling

**DOI:** 10.1186/2045-8118-10-36

**Published:** 2013-12-27

**Authors:** Shinya Yamada, Mitsue Miyazaki, Yuichi Yamashita, Cheng Ouyang, Masao Yui, Masao Nakahashi, Seiko Shimizu, Ikuo Aoki, Yukuo Morohoshi, James Gordon McComb

**Affiliations:** 1 Department of Neurosurgery, Toshiba Rinkan Hospital, Kanagawa, Japan; 2 Toshiba Medical Research Institute USA, Inc., 706 N. Deerpath Drive, Vernon Hills, Illinois 60061, USA; 3 Toshiba Medical Systems Corp., Tochigi, Japan; 4 Department of Radiology, Tokai University Oiso Hospital, Kanagawa, Japan; 5 Division of Neurosurgery, Children’s Hospital Los Angeles, Department of Neurological Surgery, Keck School of Medicine, University of Southern California, Los Angeles, CA, USA

**Keywords:** CSF, CSF movement, Respiratory movement, Balanced steady-state free precession (bSSFP) cine, Driving force of CSF

## Abstract

**Background:**

Magnetic resonance imaging (MRI) cardiac gated phase contrast (PC) cine techniques have non-invasively shown the effect of the cardiac pulse on cerebrospinal fluid (CSF) movement. Echo planar imaging (EPI) has shown CSF movement as influenced by both cardiac pulsation and respiration. Previously, it has not been possible to visualize CSF movement in response to respiration non-invasively. The present study was undertaken to do so.

**Methods:**

The effect of respiration on CSF movement was investigated using a non-contrast time-spatial labeling inversion pulse (Time-SLIP) with balanced steady-state free precession (bSSFP) readout. CSF movement was observed in the intracranial compartment in response to respirations in ten normal volunteers. To elucidate the respiration effect, the acquisition was triggered at the beginning of deep inhalation, deep exhalation and breath holding.

**Results:**

By employing this respiration-induced spin labeling bSSFP cine method, we were able to visualize CSF movement induced by respiratory excursions. CSF moved cephalad (16.4 ± 7.7 mm) during deep inhalation and caudad (11.6 ± 3.0 mm) during deep exhalation in the prepontine cisternal area. Small but rapid cephalad (3.0 ± 0.4 mm) and caudad (3.0 ± 0.5 mm) movement was observed in the same region during breath holding and is thought to reflect cardiac pulsations.

**Conclusions:**

The Time-SLIP bSSFP cine technique allows for non-invasive visualization of CSF movement associated with respiration to a degree not previously reported.

## Background

For quite some time, it has been known that CSF movement results from the formation of new CSF and motion of cilia on the surface of the choroid plexus and ependyma lining the ventricles. In the last three decades, MRI cardiac-gated PC cine techniques have been widely applied to study CSF movement non-invasively in the human brain. Using PC cine, CSF “to and fro” movement can be observed by acquiring multiple-cine phases during a cardiac-gated acquisition [1-7]. Since the observation of “to and fro” movement is associated with cardiac-gating, cardiac pulsation was thought to be the main contributor to CSF movement and to be synchronized with the cardiac beat [1,3,4,6,7]. Driving forces of CSF movement other than cardiac pulsation were thought to be minimal. CSF flow observed with the PC cine technique represents temporally averaged “to and fro” flow in a region of interest. Although each individual acquisition of the PC cine technique is acquired over a single cardiac cycle, lines of data from multiple cardiac cycles, often as many as 128 data samplings, are required to construct one image. This limitation prevents PC cine from being used to investigate fast temporal changes in CSF flow that might be seen with respiration.

A relationship between the cardiac and the respiratory influence on CSF flow has been investigated using echo planar imaging (EPI) [8-10] and one-dimensional real time acquisition and evaluation of pulsatile blood flow (RACE) techniques [11]. The EPI technique provides data acquisitions with high temporal resolution but is limited in spatial resolution. The in-plane resolution of over 3 mm × 3 mm was used in EPI as a typical matrix of 64 × 64 over a 200 mm field of view (FOV). As a result, cardiac generated pulsatile flow noted in the aqueduct of Sylvius and spinal canal varies during the different phases of respiration as it is not possible to separate the effect of these two forces moving CSF. The limitation in spatial resolution of the EPI technique reduces the ability to visualize CSF flow. The RACE technique without phase-encoding only provides signal changes over a relatively longer time period with the data display a signal graph. This method does not allow direct visualization of CSF in the format of a two-dimensional image.

The cardiac-gated, non-contrast MRI spin labeling, time-spatial labeling inversion pulse (Time-SLIP) with single-shot fast spin echo (SS-FSE) technique has been used to non-invasively observe CSF movement [12]. The Time-SLIP SS-FSE technique has a higher signal-to-noise ratio (SNR) than the PC cine technique, thereby enabling fast single-shot acquisition to improve temporal resolution without the multi-averaging required using the PC technique. In addition, this technique provides much higher spatial resolution compared to the previously reported EPI method [8-10]. Because PC cine MRI uses view sharing cardiac synchronization, PC cine MRI not only reconstructs 1 image, but can reconstruct more than 30 images in about 20 seconds during the cardiac cycle to investigate CSF velocities change along over the cardiac cycle. The technique using Time-SLIP with SS-FSE provides better temporal resolution (about 720 ms per image) than the PC cine technique (around 4 seconds per image) and thus enables one to visualize CSF movement. In this report, in order to continuously visualize CSF movement, we have improved the Time-SLIP technique using a respiratory-induced bSSFP cine with temporal resolution around 150 ms per image that allows continuous visualization of CSF movement. With this modified cine technique, we non-invasively investigate the respiratory driving force on CSF motion under normal physiological conditions that in turn will lead to a better understanding under abnormal conditions.

## Materials and methods

### Materials

We have developed a Time-SLIP method with bSSFP to acquire cine images with adequate spatial and temporal resolution to observe rapid changes in CSF movement. The fast bSSFP readout enables the technique to capture continuous movement of CSF in a shorter temporal resolution time than the previously reported single-shot fast spin echo (SS-FSE) readout [10].

### Subjects

The institutional Review Board at Toshiba Rinkan Hospital, Kanagawa, Japan gave approval for this study and informed consent was obtained from the volunteers. The study group consisted of 10 normal healthy volunteers (8 males and 2 female; age range, 28–56 years; mean age, 39 years), who were each imaged in a supine position. The volunteers were instructed to perform maximum deep inhalation, exhalation and breath holding (breathing cessation without valsalva) during which the images were acquired. Respirations or lack thereof were monitored with a chest band detector. The respiratory and cardiac cycle was recorded and the obtained images provided the triggering phase times. Each acquisition was about 6 seconds with the subjects lying supine at rest. Each experiment was repeated three times to observe the reproducibility. Due to the magnet configuration, we were not able to conduct these studies with the subjects being in either a sitting or standing position. None of the volunteers had known pulmonary disease. MRI images of the head and spine were normal in all subjects.

### Theory

**Figure 1:**
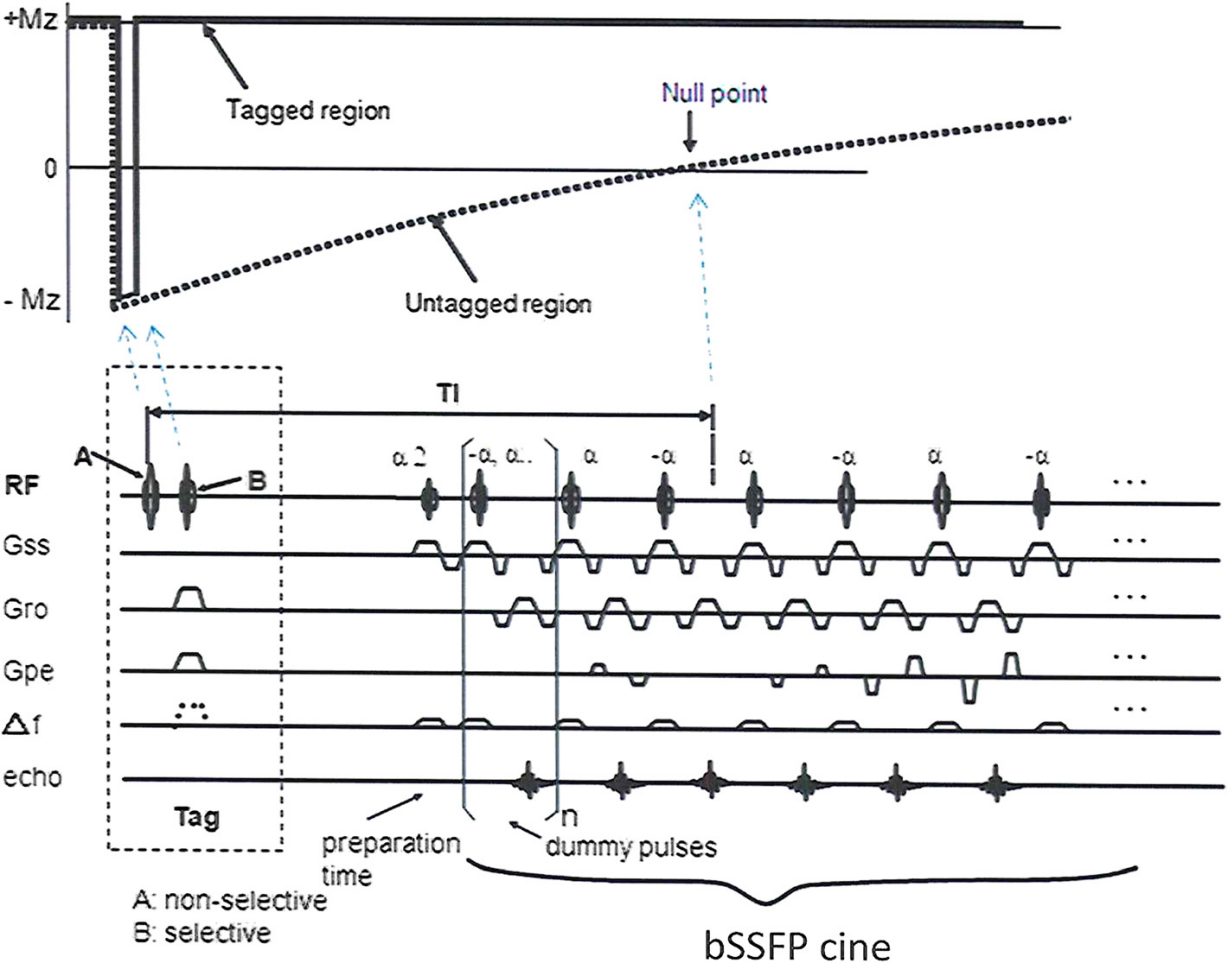
**Spin magnetization (top) and sequence diagram (bottom).** The sequence diagram (bottom) shows Time-SLIP with two-dimensional cine bSSFP. The corresponding magnetization states (top) of signals covered by the non-selective pulse **(A)** and the selective tagged pulse **(B)** are presented. An initial non-selective pulse **(A)** inverts all magnetizations within a radiofrequency coil, followed by a selective pulse **(B)** that restores the magnetization at + Mz in a plane that can be placed freely in any orientation. The untagged background signals, receiving only the non-selective inversion recovery (IR) pulse return to + Mz by following T1 recovery relaxation, as shown in the dotted line. After waiting, a TI, cine bSSFP read-out is acquired. When the untagged signals reach a null point, the tagged signals and the untagged signals have maximum contrast and thus they are well differentiated.

A flow-out technique was used in which a non-selective pulse (A) and a selective labeling (tag) pulse (B) with bSSFP cine were applied. Figure 1 shows a Time-SLIP sequence diagram with a respiration triggered bSSFP and the relationship between the regions covered by the non-selective (A) pulse and the labeling (tag) pulse (B). A non-selective inversion recovery pulse (A) inverted all signals in field of view (FOV) from the initial longitudinal magnetization (+Mz) to (−Mz). Immediately after the initial inversion, a second spatially selective B pulse was applied to invert (tag) only the magnetized tissue and fluid in the region of interest (ROI). Thus, the longitudinal magnetization in the tagged region was restored to + Mz whereas the magnetization elsewhere remained at –Mz and followed an exponential, spin lattice relaxation (T1), relaxation curve, Mz(t) = Mz(1-2exp(−t/T1). After this preparation and a variable delay period, TI, the tagged or marked CSF with the signal level of Mz(1-2exp(−TI/T1)) by the non-selective and selective tag pulses was read out by bSSFP cine. A feature of the Time-SLIP bSSFP sequence is that the region tagged by the second inversion pulse is freely selectable.

### Imaging parameters

The Time-SLIP bSSFP cine examinations were performed at Toshiba Rinkan Hospital, Kanagawa, using a 1.5-T MRI scanner (EXCELART/Vantage™; Toshiba, Tochigi, Japan), equipped with quadrature-detected -Head SPEEDER phased array coils (5 channels). The bSSFP sequence was acquired in a cine mode with adequate spatial and temporal resolution to observe motion changes of CSF movement. Typical deep respiration (inhalation and exhalation) and breath holding acquisition parameters were repetition time (TR) = 4.2 ms, echo spacing = 2.1 ms, segmentation = 1, inversion time (TI) = 230 ms, matrix = 96 (phase encoded) × 192 (read out) (352 × 384 after interpolation), slice thickness =7 mm, FOV = 24 × 26 cm^2^, parallel imaging factor = 3, and Time-SLIP tag pulse width = 10 – 30 mm. The acquired spatial resolution of 2.5 mm × 1.35 mm was interpolated to 0.68 mm × 0.68 mm. Segmentation of one means acquiring a whole 96 phase encode lines per shot. In our experiments, we achieved a temporal resolution of 134.4 ms with 40 cine phases within a single respiration in a total acquisition window of about 6 sec (134.4 ms × 40 phases = 5,376 ms). Thus, each volunteer was instructed to start deep inhalation, exhalation, or breath holding for an acquisition period of about 6 seconds directed by voice instruction. This allowed the observation of tagged CSF movement over a period from TI of 230 ms to 5.376 ms during cine acquisition. With these parameters we were able to obtain 40 images during the 6 second acquisition time. The tagged plane was placed perpendicular to the image plane allowing observation of tagged CSF movement along the image plane.

## Results

### Flow phantom validation

In order to validate the CSF flow measurement, we conducted a flow phantom study. A 4.5-mm inner diameter tube was used for the phantom and a syringe flow pump maintained a constant volumetric water flow in the tube of 0 to 9 ml/min. The flow velocity was calculated by assuming laminar flow in the tube. The tagging slab was placed perpendicular to the flow to label water spins in the tube. A continuous cine bSSFP sequence was implemented for acquisition. The entire tube was imaged in the slice direction and thus the tagged region and the movement of water was traced in the tube. From each cine image, the movement of water was calculated by a regression calculation. The movement of water using Time-SLIP cine bSSFP was plotted with the peak velocity of the laminar flow. The acquisition parameters for the flow measurement were: TR = 6.6 ms, echo spacing = 3.3 ms, matrix = 160 × 224 (320 × 448 after interpolation), slice thickness = 5 mm, FOV = 18 × 25 cm^2^, and a parallel imaging factor of 2. The acquisition window per image was 528 ms and the experiment was repeated five times. The water velocities obtained using Time-SLIP agreed with the calculated velocities (ground truth) with errors less than 1% (R^2^ = 0.9966).

### Observations of CSF movement

**Figure 2:**
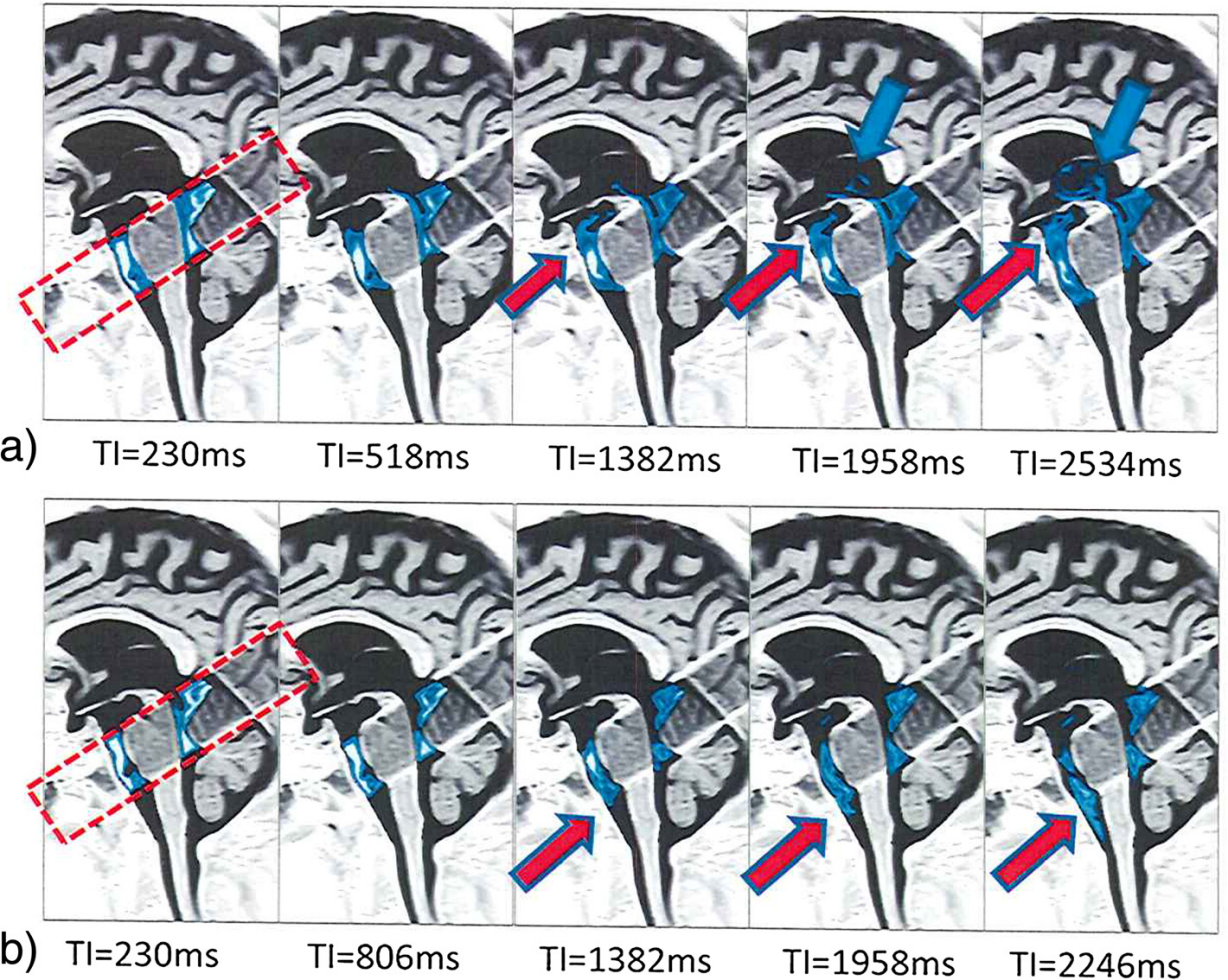
**Time-SLIP bSSFP cine images during inhalation and exhalation.** The results of the MRI cine images are shown in sagittal views of a healthy 44-year-old male volunteer. The Time-SLIP pulse was applied perpendicular to the sagittal plane to encompass the red dotted rectangle. **a)** On the sagittal image during inhalation, a caudo-cranial movement of CSF was observed at the aqueduct of Sylvius (blue arrows) and the prepontine subarachnoid space toward the suprasellar cistern and the third ventricle (red arrows). This CSF movement was noted during the initiation of inhalation lasting for about 2.5 seconds. **b)** On the sagittal image, during the period of exhalation, a cranio-caudad movement of CSF to the prepontine subarachnoid space (red arrows) was observed. Note that the movement of CSF is predominately observed in the ventral side of the subarachnoid space, which may be due to a pressure difference between the dorsal and ventral sides of the volunteer in the supine position.

**Figure 3:**
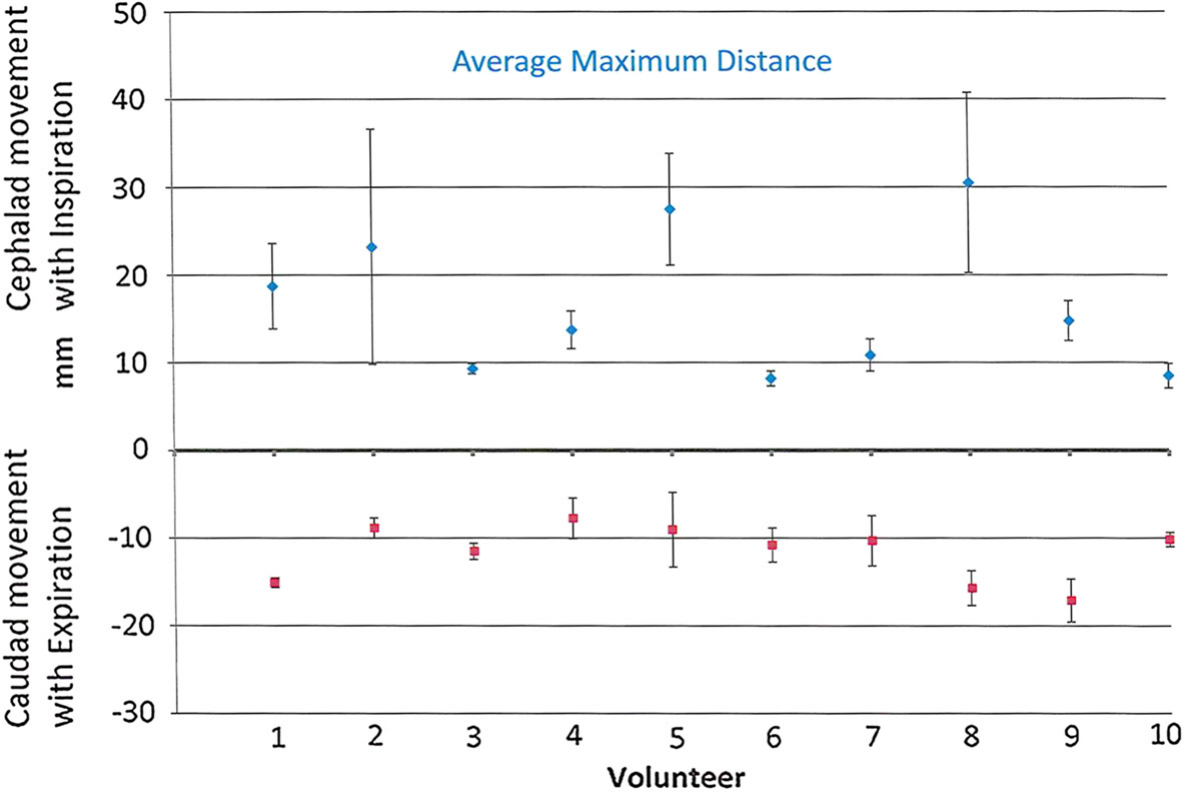
**Graph showing distance (mm) CSF in the pre-pontine cistern moved with deep inspiration and expiration.** Each of the 10 volunteers was imaged on 3 occasions. Each point on the graph shows the average maximum distance with standard deviation. For the group as a whole, CSF in the pre-pontine subarachnoid space moved cephalad 16.5 ± 7.7 mm with inspiration and caudad 11.6 ± 3.0 with expiration. Average Maximum CSF movement during deep inspiration and expiration.

The cine acquisition using Time-SLIP with bSSFP cine (Figure 1) allowed observation of continuous CSF flow, as seen in Figure 2. Figure 2a and Figure 2b show CSF flow in sagittal view during deep inhalation and exhalation, respectively. Additional file 1 and Additional file 2 are corresponding cine modes of Figure 2a and 2b, respectively. Complete sequential CSF flow can be observed with this technique. During the early phase (up to 2.5 sec) of deep inhalation, cephalad CSF flow occurred in the ventricular system and in the subarachnoid space. In the subarachnoid space, cephalad CSF flow was also observed in the prepontine cistern toward the suprasellar cisterns, as shown in Figure 2a. Caudad CSF flow occurred in the ventricular system and in the subarachnoid space during the early phase of exhalation. A substantial volume of CSF moved from the prepontine cisterns toward the ventral spinal subarachnoid space, as shown in Figure 2b. The pattern and amplitude of CSF movement associated with deep respiration observed by Time-SLIP with bSSFP cine were consistently repeatable in each individual. From the cine images the most distant CSF phase image was selected for each study and CSF movement was measured from the initial tag position at the start to the furthest CSF movement as the end point. Figure 3 shows an average maximum CSF movement during deep inspiration and expiration of 10 volunteers. The maximum distance CSF moved during deep inhalation and deep exhalation on mid sagittal image in the prepontine region was 16.4 ± 7.7 mm cephalad and 11.6 ± 3.0 mm caudad (mean+/− SD; n = 10), respectively.

**Figure 4:**
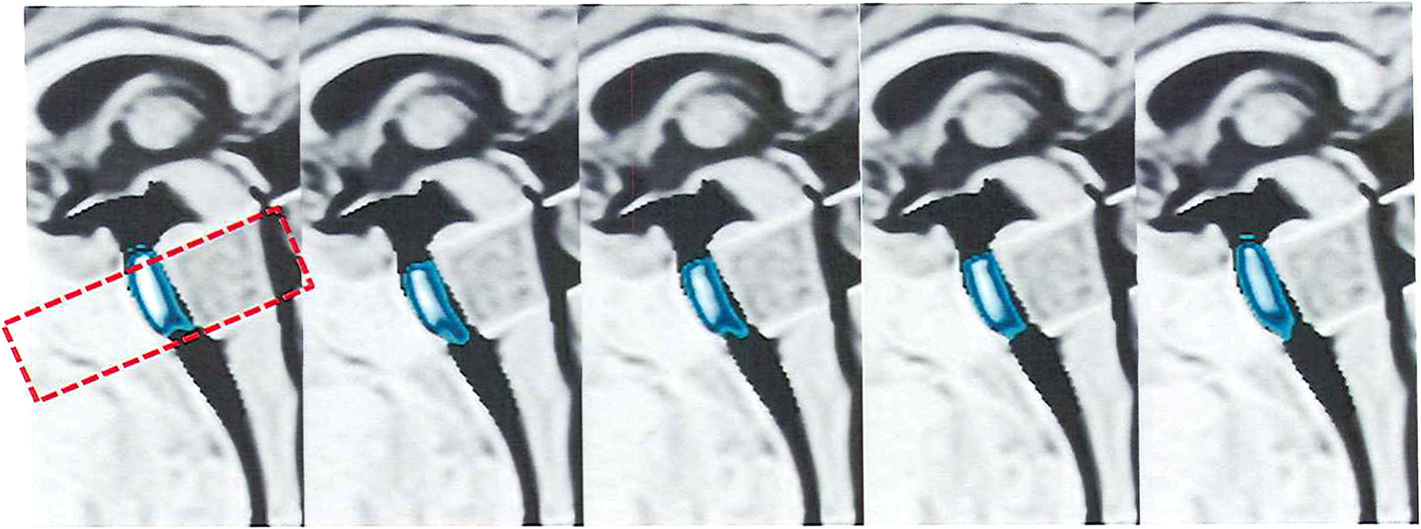
**Time-SLIP bSSFP cine images during breath holding.** The Time-SLIP pulse was applied perpendicular to the sagittal plane to encompass the red dotted rectangle. In the prepontine region, only a small amount of cephalad and caudad CSF movements were observed in the mid sagittal view. The color scale was used as an arbitrary signal intensity to highlight CSF movement. With breath holding, the small to and fro CSF movement is produced by cardiac pulsations.

**Figure 5:**
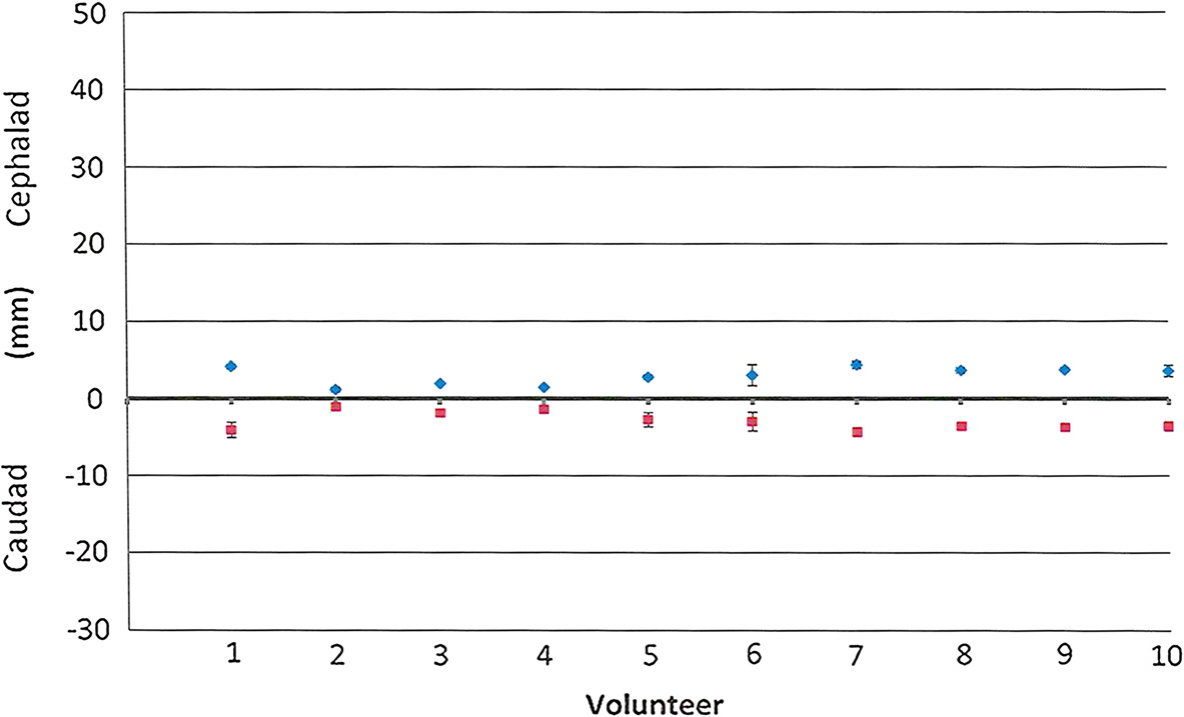
**Graph showing distance (mm) CSF in the pre-pontine cistern moved with breath holding.** Each of the 10 volunteers was imaged on 3 occasions. Each point on the graph shows the average maximum cephalad and caudad distance with standard deviation that CSF moved in the pre-pontine subarachnoid space during one cardiac cycle and is thought to reflect the systolic and diastolic components of the cardiac cycle. For the group as a whole, CSF in the pre-pontine subarachnoid space moved cephalad 3.0 ± 0.4 mm and caudad 3.0 ± 0.5 mm. CSF movement during breath holding.

Figure 4 shows a mid-sagittal view during breath holding. Small but rapid cephalad and caudad CSF volume movements were observed during breath holding. Additional file 3 shows Figure 4 in a cine mode. For breath holding, the average cephalad movement was 3.0 ± 0.4 mm and the average caudad movement was 3.0 ± 0.5 mm with a net distance of 6.0 mm (mean +/− SD; n = 10), which are summarized in Figure 5.

The *p* values (student’s *t*-test) for maximum CSF movement with deep inhalation and deep exhalation are significant when compared to breath holding, *p* < 0.001 and *p* < 0.001, respectively.

## Discussion

Our results demonstrate that a substantial amount of CSF movement occurs with deep respiration. During the deep inhalation, significant cephalad CSF movement was observed; whereas, a corresponding caudad CSF movement was noted during deep exhalation. The breath holding data showed a small but rapid to and fro CSF movement. From these observations, there is no reason to believe that “normal breathing” would show CSF movement anything but somewhere between the deep inhalation/exhalation and breath holding.

Recently, cardiac-gated PC with bSSFP has been reported to provide better CSF flow quantification by overcoming the intrinsic issues of lower SNR and temporal resolution PC with incoherent gradient echoes [13]. The cardiac-gated PC with bSSFP allows quantification measurement of CSF movement during a cardiac cycle of about 1 second, whereas Time-SLIP bSSFP cine provides CSF movement from 230 ms to over 5000 ms. Time-SLIP bSSFP cine technique also appears to be able to differentiate between cardiac and respiratory effects on CSF movement by performing the expiration, inspiration and breath holding experiments separately. Nevertheless, some cardiac pulsation effect is incorporated during deep inspiration and expiration. In addition, the tagged CSF becomes a visualizable endogenous tracer in Time-SLIP bSSFP cine, which differentiates the marked CSF movement from the background. Furthermore, post-processing image steps, averaging of signals and temporal smoothing, are not necessary. In other words, the acquired raw cine images by themselves can demonstrate CSF motion patterns precisely without additional image processing. Thus, this technique provides an easy way to readily study the CSF dynamics in any patient.

The limitation of this technique is in the temporal resolution or acquisition window of 6 seconds required for either deep inhalation or exhalation, whereas, “normal breathing” only requires about 2 to 3 seconds. This difference reflects an intrinsic delay of several seconds between respiration and CSF movement. Therefore, this method is limited to the deep respirations. Further improvement in cine temporal resolution are required to record in real time the effect of various breathing patterns on CSF movement.

A finding of this study was the significant caudad CSF movement during deep exhalation with a corresponding cephalad CSF movement during deep inhalation that visually confirms the Monro-Kellie principle. During deep inhalation, thoracic pressure diminishes followed by negative atrial pressure, resulting in increased return of venous blood from the brain. Cephalad CSF movement may occur to compensate for the blood volume reduction in the brain. Cephalad CSF movement during deep inhalation was observed in both the ventricular system and cranial and spinal subarachnoid spaces. During deep exhalation, intrathoracic pressure increases, followed by positive atrial pressure, resulting in a decrease in the venous return from the brain. Caudad CSF flow may occur to compensate for the blood volume increase in the brain. No CSF pressure measurement was performed in this study to confirm the cause of CSF movement. An advantage of this technique is that by tagging CSF it becomes an endogenous tracer allowing non-invasive visualization of CSF movement under undisturbed conditions. The relationship between brain and spinal venous volume changes during deep respiration and its influence on CSF movement need to be investigated in future studies [8,11].

Du Boulay *et al.* in 1966 conducted CSF flow observations using oil contrast medium during pneumoencephalography in humans [14]. They took exception with Bering’s theory that only the choroid plexus arterial pulsation drives CSF and instead concluded that it is the bilateral thalamic movement that is pumping CSF. Enzmann took Du Boulay’s concept a step further and concluded that it is the movement of the entire brain in response to arterial pulsation that propels CSF [5]. CSF flow associated with respiration is not at variance with this observation, only that the magnitude of CSF movement during deep inhalation/exhalation exceeds that of arterial pulsation.

CSF flow studies in animals are usually conducted with the animal in a prone position under general anesthesia. Likewise, MRI CSF flow studies in humans are done in a supine position, the one exception being a few sitting position studies using a unique MRI machine [15]. Our studies were all conducted in a supine position and showed turbulent flow and mixing of CSF in the third and fourth ventricles in response to respiration. One might suppose the degree of CSF movement in response to normal respiration should be somewhere between that of deep respirations and breath holding. CSF movement caused by normal respiration and with individuals in sitting, standing, or head down position need to be investigated as well.

## Conclusions

In summary, we have successfully shown significant CSF movement with deep respiratory excursions. The respiration-induced non-contrast MRI spin labeling, Time-SLIP with bSSFP cine provides non-invasive observation of continuous CSF movement in both the intracranial and intraspinal compartments in response to deep respirations and breath holding in normal volunteers. A significant movement of CSF was observed cephalad during deep inhalation and caudad during deep exhalation. The visualization of CSF dynamics in sequential cine views may provide valuable information and management guidance for patients with pathophysiologic conditions.

## Abbreviations

PC: Phase contrast; RACE: Real time acquisition and evaluation of motion; Time-SLIP: Time-spatial labeling inversion pulse; SS-FSE: Single-shot fast spin echo; bSSFP: balanced steady-state free precession; EPI: Echo planar imaging; SNR: Signal to noise ratio; CSF: Cerebrospinal fluid, IR: Inversion recovery; FOV: Field of view; ROI: Region of interest; T1: Spin lattice relaxation; TI: Inversion time; TR: Repetition time.

## Competing interests

There are no financial disclosures or ethnical adherences. Seven authors (MM, YY, CO, MY, MN, SS, and IA) are employees of Toshiba Medical Systems Corporation. The other authors, who are not employees of Toshiba Medical Systems Corporation, had sole control over the inclusion and evaluation of the study data and any other information that might pose conflict of interest.

## Authors’ contributions

SY, MM and YM conducted study concept and design. SY, MM, YY, CO, MY, MN, SS, IA, MY, and JGM performed data acquisition. SY, YY, and JGM analyzed and interpreted data result. SY, MM, and JGM wrote the manuscript. All authors revised the manuscript and approved the final manuscript.

## Supplementary Material

Additional file 1 **CSF flow in a sagittal view during deep inhalation.** During the early phase (up to 2.5 seconds) of deep inhalation, cephalad CSF flow occurs in the ventricular system and in the subarachnoid space. In the subarachnoid space, cephalad CSF flow was also observed in the prepontine cistern toward the suprasellar cisterns. Click here for file

Additional file 2 **CSF flow in a sagittal view during deep exhalation.** Caudad CSF flow occurs in the ventricular system and in the subarachnoid space during the early phase of exhalation. A substantial volume of CSF moves from the prepontine cisterns toward the ventral spinal subarachnoid space. Click here for file

Additional file 3 **Mid sagittal view during breath holding.** Small but rapid cephalad and caudad CSF volume movements were observed during breath holding. Click here for file
